# Synthesis and photochemical modification of monolayer thin MOF flakes for incorporation in defect free polymer composites†

**DOI:** 10.1039/d3ra04530g

**Published:** 2023-09-13

**Authors:** Karen D. J. Hindricks, Jessica Erdmann, Celine Marten, Timo Herrmann, Peter Behrens, Andreas Schaate

**Affiliations:** a Institute of Inorganic Chemistry, Leibniz University Hannover Callinstr. 9 30167 Hannover Germany andreas.schaate@acb.uni-hannover.de; b Cluster of Excellence PhoenixD (Photonics, Optics and Engineering – Innovation Across Disciplines) Welfengarten 1A 30167 Hannover Germany; c Laboratory of Nano and Quantum Engineering Schneiderberg 39 30167 Hannover Germany

## Abstract

Metal–organic frameworks (MOFs) with benzophenone linker molecules are characterized by their ability to undergo photochemical postsynthetic modification. While this approach opens up almost unlimited possibilities for tailoring materials to specific applications, the processability of the large particles is still lacking. In this work, we present a new approach to fabricate micro flakes of the stable Zr-bzpdc-MOF (bzpdc = benzophenone-4-4′-dicarboxylate) with a thickness of only a few monolayers. The crystalline and nanoporous flakes form dispersions in acetone that are stable for months. Embedding the flakes in polymer composites was investigated as one of many possible applications. Zr-bzpdc-MOF micro flakes were decorated with poly(dimethylsiloxane) (PDMS) *via* a photochemical postsynthetic modification and incorporated into silicon elastomers. The PDMS functionalization allows covalent cross-linking between the MOF and the polymer while maintaining the porosity of the MOF. The resulting hybrid materials provide defect-free interfaces and show preferential adsorption of CO_2_ over CH_4_, making them attractive for gas separation or sensing applications. The work should serve as a basis for bringing bzpdc-MOFs into real-world applications – in polymeric membranes, but also beyond.

## Abstract 2

Monolayer thin flakes of a photoreactive Zr-based MOF with benzophenone linker molecules are synthesized. Subsequent photochemical modification allows the incorporation into defect-free polymer composites showing preferred adsorption of CO_2_ over CH_4_.

## Graphical abstract

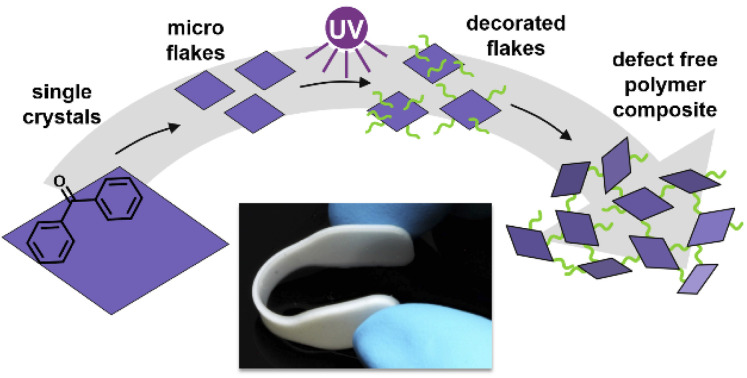

## Introduction

Metal–organic frameworks (MOFs) are characterized by their modular design from organic linkers and inorganic building units (IBUs), leading to versatile materials with tuneable pore sizes and functionalities.^1^ The adaptable properties point to numerous potential applications^2–4^ such as separation,^5,6^ gas storage,^7^ catalysis^8^ or in (opto)electronic devices and chemical sensors.^9,10^ However, MOFs remain difficult to process, which still limits their widespread use. In order to facilitate realistic applications, the combination of MOFs with functional materials has been established. By creating multifunctional composites, new materials with properties superior to those of the individual components can be designed^11–14^ using active species such as metals,^15,16^ carbon nanotubes^17,18^ and polymers.^19,20^ For the latter, several approaches have been explored by research groups: polymers grafted from and through MOF particles,^21–23^ polymers templating MOF growth,^24,25^ as well as the synthesis of MOFs using polymer ligands^26^ and the fabrication of mixed matrix membranes (MMMs).^20,27^

Recent studies have focused on the design of MOF-based MMMs for gas^28^ and liquid separation.^29^ In order to exploit the full potential of MMMs, ideal membranes are required in which the gas flows exclusively through the MOF particles. Defects at the MOF–polymer interface – such as interfacial voids, rigidification of the polymer around the filler particles, and blockage of filler pores – reduce the selectivity and must be avoided.^30^ Therefore, several approaches have been investigated to improve the dispersibility and the particle–polymer interface. First attempts focused on improving MOF–polymer interactions by introducing functional groups to the linker molecules^31–35^ or by grafting polymers onto MOF surfaces.^23,36,37^ An even more efficient way to create defect-free MOF–polymer interfaces is to create covalent bonds between filler and polymer instead of just dispersing MOF particles in polymer binders.^20^ Zhang *et al.*^38^ first introduced a covalent MOF–polymer linkage *via* a postsynthetic polymerization. Amino functionalized UiO-66-NH_2_ was modified and incorporated into a butyl methacrylate matrix. Further examples followed in which UiO-66 derivatives were post-synthetically modified and subsequently embedded in polymeric membranes.^37,39–42^ Despite the promising approach, examples with other MOFs remain rare.^43^ The reason for this is probably the high requirements for suitable MOFs: high stability,^44^ good processability^45^ and suitability for postsynthetic modification.^46^ Although many MOFs meet at least one of these requirements, very few combine all of these properties. Recently, Bury and co-workers reported a new approach that involves functionalizing IBUs rather than organic linkers for radical cross-linking in composites. A radical initiator was coordinated to open metal sites in Nu-1000 and MOF-808 *via* a postsynthetic insertion reaction.^47^ In the present work, a radical mechanism is also used, even though the initiator is integrated in the linker molecule.

We introduce a zirconium-based MOF with benzophenone moieties as new multifunctional material for the fabrication of defect-free polymer composite materials. The Zr-bzpdc-MOF, based on the angled linker molecule 4,4′-benzophenone-dicarboxylate, provides several remarkable features. Among them, the possibility for postsynthetic photochemical modifications at the keto-group.^48^ Upon excitation with UV light, the linker theoretically reacts with any compound containing a C–H bond *via* a radical mechanism.^49,50^ We recently demonstrated, that such reactions can be selectively performed at the outer and/or inner crystal surfaces – just by the choice of the modification agent.^51^ In the present work this reaction is extended to decorate the surface of Zr-bzpdc-MOF particles with functional groups and thus enabling a covalent crosslinking with polymeric matrices. Due to the outstanding customizability of bzpdc-MOFs, this paves the way to design multifunctional and highly tuneable MOF membranes for a wide range of applications.

However, to date, bzpdc-MOFs are only available as large single crystals or thin films,^48,52^ which are unsuitable for incorporation into polymers. Therefore, as a first step we investigated a synthesis route towards ultrathin Zr-bzpdc-MOF micro flakes. Resulting MOF particles with a thickness of only a few monolayers form stable dispersions over month and are well dispersible in polymers. In a second step, the micro flakes were incorporated into poly(dimethylsiloxane) (PDMS) hybrid materials. PDMS is one of the most attractive and widely used polymers for gas separation membranes.^53^ The polymer readily forms thin membranes and shows high permeability^54^ but the pure dispersion of MOF particles in PDMS results in pore blocking and plugged-sieve effects (especially at high MOF loadings).^55–57^ Cohen *et al.* reported covalent crosslinking of MOFs in PDMS for the first time. An allyl-substituted derivative of the UiO-66 MOF was grafted with PDMS increasing the dispersibility, compatibility and integration of the particles in the polymer.^40^ We chose a similar approach and attached the two siloxanes poly(dimethylsiloxane) (PDMS) and hydride terminated poly(dimethylsiloxane) (PDMS-H) to the bzpdc-linker *via* the easily accessible photochemical modification. Resulting surface modifications enable the covalent cross-linking with the silicone elastomer SYLGARD® 184 and thus defect free MOF–polymer interfaces. Hybrid materials with filler contents up to 30% were prepared.

PDMS was selected as a model polymer for this study. However, other polymers may be advantageous depending on the intended application. In general, rubbery polymers such as PDMS exhibit high permeabilities but lack selectivity, while glassy polymers offer good separation performance but low throughput.^58^ A variety of polymers have been utilized in recent years to create MOF–polymer hybrid materials with different form factors to match the intended application.^20,59^ The approach presented here should be readily transferable to other (polymeric) materials in future research, allowing the production of MOF composites with tailored properties for specific applications.

## Experimental

### Materials

Following chemicals were used without further purification: acetone (99.5%, Roth), benzophenone-4,4′-dicarboxylic acid (95%, abcr, H_2_bzpdc), dichloromethane (99.8%, Sigma-Aldrich, DCM), *N*,*N*′-dimethylformamide (99.8%, Sigma-Aldrich, DMF), ethanol (absolute, EMPLURA®), formic acid (98%, Merck, FA), trimethylsiloxy terminated poly(dimethylsiloxane) (thermo scientific, PDMS, Mw410), hydride-terminated poly(dimethyl-siloxane) (Sigma-Aldrich, PDMSH, Mw580), zirconyl chloride octahydrate (98%, Sigma-Aldrich, ZrOCl_2_·8H_2_O), zirconium(iv)-chloride (99.5%, Sigma-Aldrich, ZrCl_4_) and SYLGARD® 184 (Sigma-Aldrich).

### Synthesis of Zr-bzpdc-MOF micro flakes (1)

For the synthesis of Zr-bzpdc-MOF micro flakes 1, a comprehensive parameter screening was carried out, starting from our published single crystal synthesis.^48^ The successful reflux synthesis of Zr-bzpdc-MOF micro flakes is possible according to the following recipe: 0.119 g ZrCl_4_ (0.51 mmol) were dissolved in 10 mL DMF under stirring at 130 °C. A clear solution of 1.95 mL (51.2 mmol) formic acid and 0.277 g (1.02 mmol) H_2_bzpdc in 10 mL DMF (preheated to 130 °C) was quickly added and the reaction mixture was stirred for 72 h at 130 °C. After about 30 minutes, the reaction mixture became opaque. After the reaction, the resulting white solid was centrifuged, washed with DMF and ethanol and dried under reduced pressure (yield 140 mg).

A tenfold batch of Zr-bzpdc-MOF micro flakes synthesis was performed according to the procedure described above. The reaction was performed in a 500 mL round bottom flask at 130 °C for 144 h. The resulting white powder was thoroughly washed twice with DMF (100 mL), three times with ethanol (100 mL) and dried in air (yield = 1.5 g).

### Downsizing of Zr-bzpdc-MOF micro flakes (2)

Zr-bzpdc-MOF micro flakes were dispersed using a Branson 450 Digital Sonifier ultrasonic finger. Acetone was found to be the most suitable dispersion medium. The Zr-bzpdc-MOF micro flakes (1) were dispersed in 80 mL of acetone in a glass vessel with a screw cap and treated with ultrasound at 50% power for 1 h with stirring and external cooling with ice water. The solid was then separated from the solvent by centrifugation (5000 rpm, 10 min) and dried under reduced pressure. To check the influence of the ultrasonication power, tests were also carried out at 30% and 40% power. At power levels higher than 50%, the solution heated up too much.

### Synthesis of Zr-bzpdc-MOF single crystals

Zr-bzpdc-MOF single crystals were synthesized as reference material according to a previously published procedure.^48^ 3.22 g (10 mmol) ZrOCl_2_·8H_2_O and 5.40 g (20 mmol) H_2_bzpdc were dissolved in 200 mL DMF. After adding 56.59 mL (1500 mmol) of formic acid, the clear solution was transferred to a screw-cap glass vessel with Teflon-sealing and kept at 120 °C for 10 days in a circulating air oven. The resulting colourless powder was washed extensively with acetone, dried on air and purified *via* Soxhlet extraction in acetone for 12 h.

### Photochemical postsynthetic modification of Zr-bzpdc-MOF micro flakes

For postsynthetic modification of 2, 600 mg of the product were dispersed in 6 mL of the respective modifying reagent (PDMS or PDMSH) and transferred to a UV transparent quartz tube. In order to exclude oxygen, the setup was flushed with Ar for 15 min and subsequently sealed. The modification was performed by irradiation with a 365 nm UV-LED Spot (5 W) from Lumimax® at a distance of 4 cm and an irradiation time of 20 h. To remove unbound molecules, the product was centrifugated, washed with acetone and stirred overnight in DCM (25 mL) before dried under reduced pressure. Resulting modified Zr-bzpdc-MOF micro flakes are named 2-PDMS and 2-PDMSH.

### Preparation of polymer composites

Pristine crushed Zr-bzpdc-MOF micro flakes (2) as well as modified flakes 2-PDMS and 2-PDMSH were used for the incorporation into silicone elastomers with different filler contents. The polymer used is the two-component system SYLGARD® 184 with a mixing ratio of the two components (designated as comp-A and comp-B) of 10 : 1. To improve the dispersibility the solid was sieved (mesh size 200 μm) before further use. The mass fractions and the masses of comp-A and comp-B, as well as the micro flakes, are listed in Table S1 in the ESI.† A total weight of 375 mg was chosen for all samples. To prepare the polymer mass, comp-A was placed in a polypropylene screw-capped jar and the Zr-bzpdc-MOF micro flakes were added. The mixture was homogenized in a Hauschild SpeedMixer® DAC150 SP, mixed with comp-B and homogenized again. The viscous mass was then transferred to Teflon moulds and evenly distributed. Air bubbles in the mass were removed under vacuum (30 min) before the hybrid materials were cured at 80 °C in a circulating air oven for 24 h. After the first 30 min, lids were mounted to ensure a homogeneous thickness of the samples. After cooling to room temperature, the elastic rubber-like samples composite-2, composite-2-PDMS and composite-2-PDMSH were easily removed from the Teflon moulds and used for further characterization. For comparison, also samples without MOF-filler were prepared, denoted as pure silicone.

### Characterization techniques

X-ray diffraction (XRD) measurements of Zr-bzpdc-MOF powder samples were performed on a STOE STADI-P transmission diffractometer equipped with a DECTRIS MYTHEN 1K silicon strip detector. Monochromatization of CuKα1 radiation (*λ* = 1.540594 Å) was realized by a curved Ge(111) monochromator crystal which was placed in the primary beam. Measurements were performed with a step size of 1.5° 2*θ* between 2 and 50° 2*θ* and a measurement time of 10 s per step. The powder was fixed between two X-ray amorphous foils. X-ray diffraction measurements of Zr-bzpdc-MOF composite materials were performed on a STOE X-ray diffractometer operating in Bragg–Brentano geometry. An Iso-Debyeflex 3003 was used for the generation of X-rays, delivering CuKα1 radiation (*λ* = 1.540594) which was monochromatized with a graphite monochromator placed in the secondary beam. Measurements were performed with a step size of 0.015° 2*θ* and a measuring time of 7 s per step between 4 and 12° 2*θ*.

Sorption measurements were performed on Micromeritics instruments with different gases on both the powder materials and the composite materials (cut into small pieces). For data analysis the software Microactive was used. Prior to the measurement, all samples were activated at 50 °C under secondary vacuum for 20 h. Argon physisorption measurements were performed at 87 K on a 3Flex instrument. During the measurement, the saturation pressure *p*_0_ was determined in a reference tube for each isotherm point. The Brunauer–Emmett–Teller (BET) equation was used to determine the surface-area in the range of *p*/*p*_0_ = 1 × 10^−4^ to 1 × 10^−2^. For CO_2_ and CH_4_ sorption at 25 °C, the ASAP 2020 instrument was used. Scanning electron microscopy (SEM) images were taken on a Hitachi Regulus 8230 at 0.5 kV and a working distance of 4 mm. Cross-sectional images were taken at 5 kV and a working distance of 8 mm. A FEI Tecnai G2F20 TMP in brightfield mode was used for transmission electron spectroscopy (TEM). Samples of Zr-bzpdc-MOF micro flakes were prepared from a dispersion in acetone on a 400 mesh carbon-coated Cu grid. Evaluation was performed using the software ImageJ from Fiji. Atomic force microscopy (AFM) investigations were performed on a Park systems NX10 in non-contact mode under ambient conditions. Zr-bzpdc-MOF micro flakes were dispersed in acetone and the suspension was dropped onto a silicon wafer. For liquid phase ^1^H nuclear magnetic resonance spectroscopy (^1^H-NMR) 10 mg of the powdered samples were dissolved in a mixture of 100 μL DCl (35 wt% in D_2_O) and 600 μL DMSO-d6. Spectra were measured on a BRUKER Ascent instrument at 400 MHz and were analysed using MestReNova software. Infrared (IR) spectroscopy of powdered samples was performed on a Bruker Tensor 27 FT-IR spectrometer equipped with an attenuated total reflection (ATR) unit. Thermogravimetric (TG) data were obtained with a Netzsch STA 429 Thermoanalyzer at a heating rate of 10 K min^−1^ on synthetic air. Dynamic Light Scattering (DLS) particle size determination was performed on a Malvern Panalytical Zetasizer Nano ZS in acetone. Three measurement curves were recorded for each sample. Torsion experiments were performed on an Instron 5565A. The samples were prepared as “shoulder rods” with a length of 5 cm, a height of 1 mm and a width of 4 mm according to the ISO 37 standard. The probes were clamped vertically in the machine at the ends using two hydraulic jaws, 7 mm was clamped into the jaws at each end. The specimen was stretched at a tensile rate of 20 mm min^−1^ until it broke.

## Results and discussion

### Zr-bzpdc-MOF flakes

**Fig. 1:**
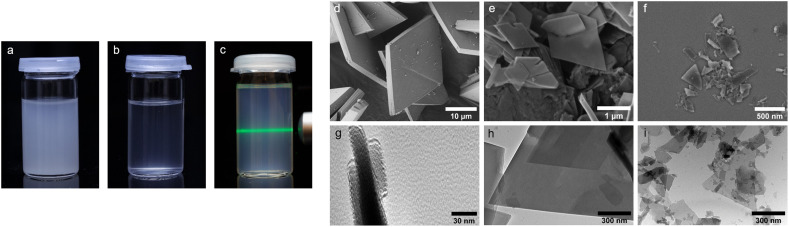
Dispersions of Zr-bzpdc-MOF micro flakes in acetone: (a) micro flakes 1 direct after preparation and (b) after 24 h; (c) downsized flakes 2 after 6 months. SEM images of Zr-bzpdc-MOF as (d) single crystals, (e) micro flakes 1 and (f) micro flakes after ultrasonication 2. TEM images of micro flakes 1 in (g) cross section and (h) top view; (i) TEM image of micro flakes after ultrasonication 2.

For the homogeneous incorporation of Zr-bzpdc-MOFs into polymer composites, a small particle size is essential. Although the Zr-bzpdc-MOF single crystals could be delaminated into thin flakes by ultrasonic treatment in isopropanol, this top-down approach lacks sufficient yield and homogeneous particle size.^48^ In general, bottom-up strategies are preferred for the downsizing of MOF particles^60^ and thus a new synthesis route was developed, based on the modulated^61^ single crystal synthesis already published by our group.^48^ Instead of the solvothermal reaction route, the synthesis was carried out under reflux and various parameters such as reaction time, temperature and precursors were systematically varied. Detailed descriptions of the synthesis series performed can be found in the ESI (ESI, Section S1†). The best results were obtained at a reaction temperature of 130 °C, a reaction time of 72 h and by using zirconium(iv) chloride as the Zr precursor. Fig. S1 in the ESI† shows the synthesis route, which can be described as a “hot injection”.^62^ Linker and the modulator formic acid as well as the Zr precursor are dissolved separately in DMF, heated to 130 °C and rapidly combined. After a short time, the solution becomes cloudy and a white solid precipitates. The synthesis is easily upscaled and the resulting product, hereafter referred to as 1, was further mechanically downsized by ultrasonic treatment. Crushed particles are denoted as 2. Best results were obtained with a power of the ultrasonic finger of 50%. Lower powers show slightly worse results (see DLS measurements in the ESI Section S1.3†), while at higher powers the heat input became too high and the solution heated up too much. The treatment results in dispersions of the downsized product 2 in acetone that are still stable after several months. In comparison, 1 precipitates after several hours (Fig. 1a–c and S8†).

**Fig. 2:**
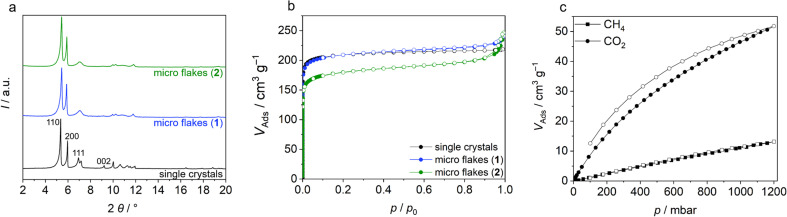
(a) PXRD and (b) argon physisorption of Zr-bzpdc-MOF single crystals, micro flakes 1 and downsized micro flakes 2. Indices of the first reflections are depicted in the PXRD; (c) CH_4_ and CO_2_ sorption experiments (298 K) with downsized Zr-bzpdc-MOF micro flakes 2. Adsorption is shown with filled symbols and desorption with empty symbols.

When scanning electron microscopy (SEM) and transmission electron microscopy (TEM) images of Zr-bzpdc-MOF single crystals from a solvothermal synthesis as well as product 1 from “hot injection” and 2 after ultrasonication are compared, a clear size reduction becomes obvious (Fig. 1d–i). While the single crystals have diameters of about 30 μm, the size of 1 is already reduced (2 to 6 μm) and a further reduction was achieved after ultrasonication (80 to 530 nm). Thereby, the overall topology of Zr-bzpdc-MOF is preserved. 1 is present as very thin rhombohedral micro flakes. After ultrasonic treatment the flakes are broken and thus reduced in size. The background of the underlying micro flakes is visible through the flakes, indicating a thickness of only a few monolayers for 1 and 2. The TEM image in Fig. 1g confirms the very low thickness of the flakes. Three agglomerated flakes with a thickness of 11 to 15 nm are shown in cross section. AFM images (ESI Fig. S9†) show mostly agglomerated flakes (analogue to SEM and TEM) as a consequence of the preparation from dispersions in acetone. Nevertheless, the steps of individual monolayers can be resolved by measuring the height profile. X-ray powder diffractograms (PXRDs) of single crystals and the micro flakes (1 and 2) (Fig. 2a) show good agreement. As result of the low expansion in the *c*-direction, the (002) reflection is extinguished in the micro flakes PXRDs, which has also been observed in delaminated sheets of single crystals.^48^ There are no changes in the X-ray powder diffractogram before and after the ultrasonication. Additionally, lattice parameters (*a* = 30 Å and *b* = 19.3 Å) of micro flakes 2 were determined *via* HR-TEM (ESI Fig. S6†) and are also in good agreement with the literature.^48^ Argon physisorption measurements (Fig. 2b) show that the porosity is also preserved. Typical I(a) isotherms are obtained for both single crystals and micro flakes. The BET areas for single crystals and micro flakes 1 are to 730 m^2^ g^−1^. The slight increase at high relative pressures is due to interparticle pores between the micro flakes (as a result of the smaller particle size). Downsized micro flakes 2 show only a slight decrease in porosity, the BET area still reaches values of 620 m^2^ g^−1^. The slightly reduced porosity is probably due to the mechanical stress during ultrasound treatment.

Overall, through the combination of (hot-injection) reflux synthesis and mechanical comminution, the synthesis of Zr-bzpdc-MOF as ultrathin micro flakes while maintaining crystallinity and porosity is possible. Compared to mechanical comminution of the single crystals, advantages such as significantly increased yield and higher homogeneity result.^48^

Further sorption experiments were performed with CO_2_ and CH_4_ measurements at 25 °C. The isotherms in Fig. 2c show a much better affinity of the pore system for CO_2_. The maximum uptake at 1 bar corresponds to 52 cm^3^ g^−1^ for CO_2_ and 13 cm^3^ g^−1^ for CH_4_, respectively, which correlates to a difference by a factor of 4. Such differences in sorption behaviour for different molecules are a prerequisite for many applications, such as sensing or gas and liquid separation.

Not least due to the ability of further post-synthetic modification of the pore system, the Zr-bzpdc-MOF thus appears to be a promising material in this context. To make the properties useable, however, further shaping is indispensable – composite materials or films are generally far superior to powdered materials in terms of their applicability.^63,64^ For this reason, the crushed flakes were incorporated into polymeric composite materials. As polymer the silicon elastomer SYLGARD® 184 is used.

### Fabrication of polymer composites

**Fig. 3:**
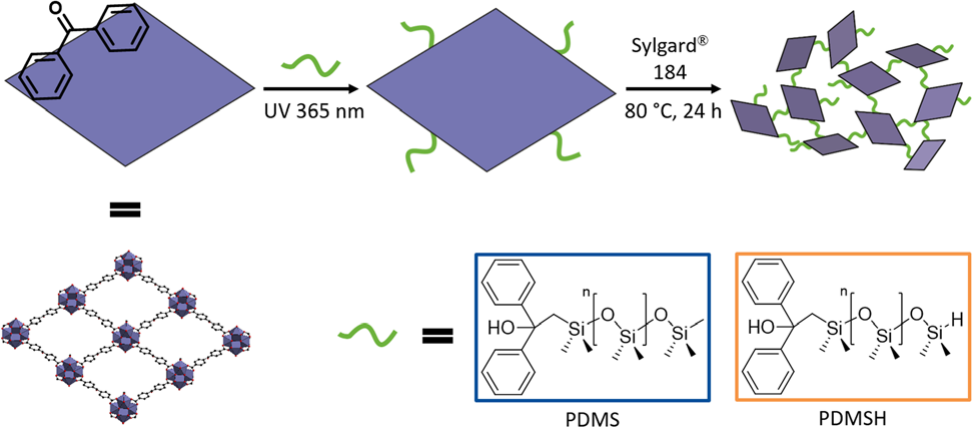
Schematic representation of the photochemical postsynthetic surface modification of Zr-bzpdc-MOF micro flakes with siloxanes and subsequent covalent crosslinking with the silicon elastomer SYLGARD® 184. Poly(dimethylsiloxane) (PDMS) and hydride terminated poly(dimethylsiloxane) (PDMSH) were used.

In order to produce defect-free composites without voids between MOF and polymer, we target a crosslinking between Zr-bzpdc-MOF micro flakes and the silicon elastomer. Taking advantage of the easily accessible photochemical modification of bzpdc-MOFs,^22,48,51,52,65,66^ the two poly(dimethylsiloxanes) PDMS and PDMSH shall be covalently bonded to the downsized micro flakes 2 in a first step. Thus, in a second step, poly(dimethylsiloxane) decorated micro flakes and the silicon elastomer could be crosslinked during the curing process. A scheme of this strategy is shown in Fig. 3. Since SYLGARD® 184 cures *via* a hydrosilylation reaction – the condensation of Si–H with vinyl moieties in presence of a catalyst^67^ – the crosslinking with hydride terminated PDMSH might be favoured. As control groups, pure silicone elastomers and composites with unmodified Zr-bzpdc-MOF micro flakes were synthesized.

#### Postsynthetic modification

**Fig. 4:**
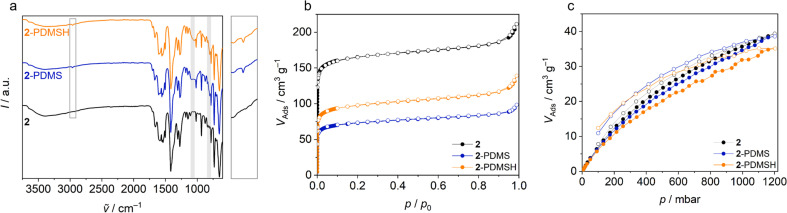
Pristine micro flakes (2) and flakes modified with PDMS (2-PDMS) and PDMSH (2-PDMSH). Plots of (a) IR spectroscopy, (b) Ar physisorption isotherms (*T* = 97 K) and (c) CO_2_ sorption isotherms (*T* = 298 K). In the IR spectra, vibrational bands assigned to covalently bonded PDMS and PDMSH are highlighted, the region around 2690 cm^−1^ (ν_–C–H_) is enlarged and shown on the right side of the plot.

For the postsynthetic modification, micro flakes 2 were dispersed in the respective siloxane and irradiated with UV light of 365 nm wavelength for 20 h in the absence of oxygen. Subsequent intensive washing with acetone and DCM and stirring overnight in DCM removed unattached molecules. Resulting modified flakes are named 2-PDMS and 2-PDMSH. PXRDs (ESI Fig. S10†) show no changes compared to the unmodified flakes 2, the crystallinity is thus preserved and there are no extensive structural changes. The modification is detectable *via* IR spectroscopy (Fig. 4a). 2-PDMS and 2-PDMSH show additional bands at 2690 cm^−1^ (ν_–C–H_), 1085 cm^−1^ (ν_Si–O–Si_) and 805 cm^−1^ (ν_Si–CH_3__), which can be assigned to the attached poly(dimethylsiloxanes).^68^ Using TG (ESI Fig. S11†), the siloxane content can be calculated to be 10.8% for 2-PDMS and 11.6% for 2-PDMSH. The detailed calculation can be found in the ESI (Section S2.1†). ^1^H NMR spectroscopy (ESI Fig. S12†) shows that the modification occurred only on the outer surface. The signal of the aromatic protons of the benzophenone linker is almost unchanged in all samples. Only small signals, caused by the binding at the outer surface, are detectable. In general, more PDMSH seems to be attached to Zr-bzpdc-MOF flakes compared to PDMS, which is analogous to the results of the TG. A more detailed discussion is part of the ESI Section S2.2.† Argon physisorption measurements (Fig. 4b) yield BET areas of 250 m^2^ g^−1^ and 330 m^2^ g^−1^ for 2-PDMS and 2-PDMSH, respectively. Due to the covalent bonding of the siloxanes to the outer surface of Zr-bzpdc-MOF micro flakes, argon adsorption is reduced. However, the modification does not affect CO_2_ adsorption (Fig. 4c). For all samples, the maximum CO_2_ uptake at 25 °C remains constant at about 40 cm^3^ g^−1^. Two reasons can be assumed for this. First, CO_2_ has a quadrupole moment and a higher kinetic energy at 25 °C, which is known to make very narrow pores accessible.^69^ If the pore windows are partially reduced by the bonding of the poly(dimethylsiloxanes), CO_2_ molecules may be able to pass better than the inert argon. On the other hand, CO_2_ has a significantly higher solubility in poly(dimethylsiloxanes).^54^ The molecule can thus adsorb in a PDMS shell, but also diffuse through it and reach the pore system of the Zr-bzpdc-MOF. Overall, the modification with the two poly(dimethylsiloxanes) PDMS and PDMSH was successful and the pore system of Zr-bzpdc-MOF remains accessible to CO_2_. In a next step, covalent crosslinking with the silicone elastomer (SYLGARD® 184) was performed.

#### Fabrication of composite materials

**Fig. 5:**
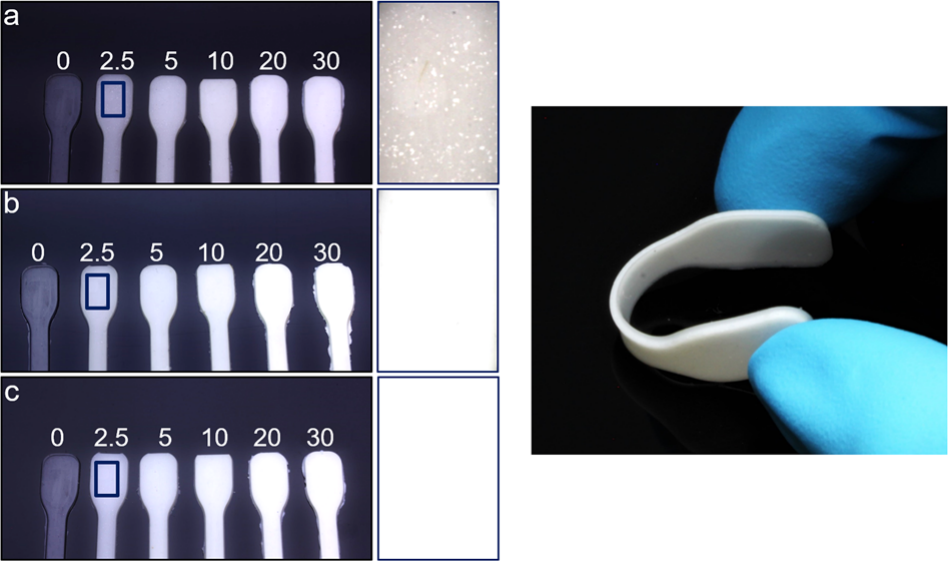
Left: photographs of composite materials with different mass fractions of Zr-bzpdc-MOF micro flakes. (a) composite-2 with pristine flakes, (b) composite-2-PDMS with PDMS modified flakes and (c) composite-2-PDMSH with PDMSH modified flakes. Enlarged images are shown on the right side for 2.5 wt% samples to assess differences in homogeneity. Right: photograph of a flexible composite.

The elastomers were prepared with mass fractions of 0 wt% to 30 wt% of pristine Zr-bzpdc-MOF micro flakes 2 as well as modified flakes 2-PDMS and 2-PDMSH. The pure silicone (SYLGARD® 184) served as control group. Photos of the resulting composite materials composite-2, composite-2-PDMS and composite-2-PDMSH are shown in Fig. 5. The samples are elastic and bend easily. However, they become somewhat stiffer as the mass fraction increases. Samples with the lowest mass fraction of 2.5% show clear differences in homogeneity. Composite-2 has agglomerated solid particles, while the modified flakes in composite-2-PDMS and composite-2-PDMSH are homogeneously distributed. The flakes are better dispersible in the silicone due to the modification. XRDs of the composites with 30 wt% are part of the ESI (Fig. S13†) and confirm that the crystallinity of the flakes is preserved. TG measurements on composites with 10 wt% and 30 wt% show no significant differences between samples with differently modified flakes.

Thus, the filler content does not affect the stability. Decomposition takes place between 300 °C and 500 °C (ESI Fig. S14†).

**Fig. 6:**
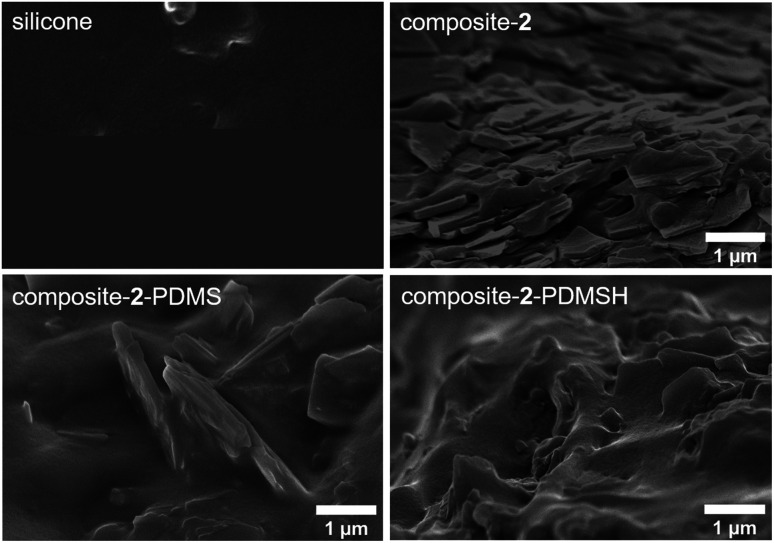
SEM images of the pure silicon (SYLGARD® 184) and composites with 30 wt% MOF content.

Fig. 6 shows SEM images of the different composites with 30 wt% MOF content and the pure silicone (SYLGARD® 184). Differences in the bonding of the silicone to the MOF particles are clearly visible. When the pure flakes are used in composite-2, there are large voids between the particles and the polymer. In contrast, composites with the modified flakes 2-PDMS and 2-PDMSH show defect-free MOF–polymer interfaces. Therefore, the modification can be considered as a success. The defect-free interfaces are a basic requirement for high performance applications *e.g.* sensing or gas/liquid separation.

In the following, it was investigated whether the improved integration of the flakes also has an effect on the sorption and mechanical properties. To determine the mechanical properties, tensile tests were performed on the composite materials. The specimens were stretched uniaxially in length at a constant tensile rate. Three measurements were taken on different specimens for each filler content. The resulting stress–strain curves and measured values are part of the ESI (Section S3.2†).

In general, it should be noted that the mechanical measurements are complex and subject to inherent variations due to the measurement setup. In addition to accurate specimen clamping and adjustment, ideal specimens must be available for 100% comparable measurements.

**Fig. 7:**
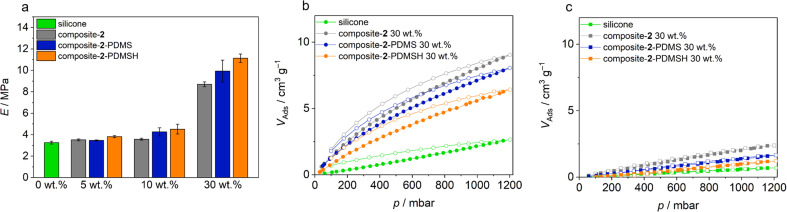
(a) Young's modulus (*E* = *σ*/*ε* with mechanical stress *σ* and strain *ε*), (b) CO_2_ sorption (298 K) and (c) CH_4_ sorption (298 K) of composites with different MOF contents. Pristine Zr-bzpdc-MOF micro flakes 2 and modified flakes 2-PDMS and 2-PDMSH were used, resulting in composite-2, composite-2-PDMS and composite-2-PDMSH. Measurements of pure silicon (SYLGARD® 184) are shown for comparison.

To compare specimens, Young's moduli were determined from the slopes of the graphs in the first 10%. In general, Young's modulus *E* increases with a material's resistance to elastic deformation and corresponds to the slope of the linear-elastic region of the stress–strain diagram from the tensile tests (*E* = *σ*/*ε* with mechanical stress *σ* and strain *ε*). Means and standard deviations for the Young's modulus *E* are shown in Fig. 7. The Young's modulus increases with increasing filler content. Solid particles in the silicone make it more difficult for the polymer chains to unravel under tension, so more force must be applied to elongate the sample. In addition, the elastic moduli of all elastomers with modified MOF particles are higher than their unmodified analogues. This confirms the observation from the SEM images that the modified micro flakes are significantly better bound in the silicone. The highest strengths are found in PHM samples containing flakes modified with PDMSH. This observation is consistent with previous results that the modification with PDMSH was more extensive than with PDMS. Another reason for the higher strength may be better crosslinking between MOF and polymer, enabled by the Si–H groups in PDMSH.

Sorption experiments complete the study of the polymer composite materials. For the measurements, 200 g of the 30 wt% composite-2, composite-2-PDMS and composite-2-PDMSH were cut into small pieces. The pure silicone (SYLGARD® 184) served as control group. Argon physisorption isotherms initially show no adsorption (ESI Fig. S15†). However, this is not to be expected due to the slow diffusion of argon through the polymer and although PDMS membranes typically have low BET surface areas, they are well suited for use as gas separation membranes.^70^ Gas permeation is based on a solution–diffusion mechanism where the gas molecules dissolve in the membrane and diffuse through the membrane due to a concentration gradient. In ideal MMMs, molecules can thus pass through the membrane and porous particles even if the BET area is low.^70,71^ Separation of the different permeants can be achieved by (a) different amounts dissolved in the membrane or (b) diffusion rates through the membrane.^72^ Typically, diffusion coefficients decrease with increasing penetrant size. At the same time, larger penetrants are more condensable and therefore more soluble than smaller ones. For rubbery polymers such as PDMS, there is usually little ability to separate molecules due to their size and solubility becomes the determining therm. This results in a higher permeability of CO_2_ compared to, for example, CH_4_ or N_2_.^54,70^

As already shown, the Zr-bzpdc-MOF shows an increased adsorption of CO_2_ compared to CH_4_. Thus, the polymeric hybrid materials should show improved CO_2_ adsorption compared to the pure silicones, while the CH_4_ sorption should remain the same. In order to prove this assumption, sorption measurements with these two gases were carried out on the composite materials. The isotherms recorded at 25 °C are shown in Fig. 7. Indeed, a preferential adsorption of CO_2_ over CH_4_ is also observed in the composites. Compared to the pure silicone elastomer (SYLGARD® 184), an increased uptake of CO_2_ can be observed in all composites, while the adsorption of CH_4_ is only slightly enhanced. Since the proportion of nanoporous MOF particles is only 30 wt%, the maximum adsorption is correspondingly reduced compared to the pure MOF flakes. A further reduction of the porosity, *e.g.* due to pore blocking effects, is not observed. Only minor differences are observed between composites with differently modified flakes. Composite-2 shows a slightly higher maximum CO_2_ uptake, followed by composite-2-PDMS and finally composite-2-PDMSH with the lowest value. Thus, the trend is opposite to the degree of modification and the quality of binding. This is to be expected because on the one hand a higher degree of modification makes the adsorption somewhat more difficult and on the other hand there is the possibility that molecules adsorb in the voids between the MOF and the polymer. This leads to an apparently higher adsorption. For the time being, it should be noted that, analogous to the previous findings, selective adsorption is possible, which in principle predestines the materials for application in sensing, gas separation and beyond.

## Conclusions

In this work, a MOF based on photoreactive benzophenone linker molecules was prepared for the first time as particles with extensions in the lower nanometre range. After intensive synthesis optimization, micro flakes of the stable Zr-bzpdc-MOF, with a thickness of only a few monolayers were obtained. Compared to their single crystal analogues, porosity and crystallinity are preserved, while the number of potential applications increases significantly due to better processability. The flakes are postsynthetically modifiable and form dispersions that are stable for months in solvents such as acetone.

As already shown for the single crystals,^48,51^ the properties of the micro flakes can be tailored by photochemical covalent attachment of modifying reagents. Almost any molecule containing a C–H bond can be attached. Here, this possibility for postsynthetic modification was used to prepare the particles for embedding in composite materials. By surface modification with poly(dimethylsiloxanes), covalent crosslinking between MOF particles and the silicon elastomer SYLGARD® 184 was realized. This avoids defects at the MOF–polymer interface as well as pore blocking effects – two basic requirements for the use of the composite materials (*e.g.* in MMMs for gas separation or in sensing devices). Additionally, the composite materials also show a preferential adsorption of CO_2_ over CH_4_.

Our results demonstrate that the Zr-bzpdc-MOF is a promising filler in (polymeric) composite materials. The advantage of post-synthetic modification, which is easy to perform, should be emphasized once again, as it opens up a variety of other possibilities:

(a) Further modification with functional groups on the linker molecules can favour adsorption of various gases to improve gas separation properties.^73^

(b) The flakes can be prepared as fillers for a wide variety of polymers or other composite materials to obtain defect-free materials by easy assessable postsynthetic modification.

(c) Stable dispersions allow further applications such as in coatings.

Thus, our work can be seen as a foundation that opens up many possibilities for using bzpdc-MOFs in real-world applications.

## Conflicts of interest

There are no conflicts to declare.

## Supplementary Material

RA-013-D3RA04530G-s001
