# Supplementary material for: Synthesis and photochemical modification of monolayer thin MOF flakes for incorporation in defect free polymer composites
Source: RSC Adv. 2023 Sep 13;13(39):27447–55. doi: 10.1039/d3ra04530g (PMC10498359; doi:10.1039/d3ra04530g)
Supplement: RA-013-D3RA04530G-s001 [file RA-013-D3RA04530G-s001.pdf]

Electronic Supplementary Material (ESI) for RSC Advances.
This journal is © The Royal Society of Chemistry 2023

Table of content

Section S1: General synthesis and parameter variation 2

S1.1 Variation of reaction time & -temperature 2

S1.2 Variation of Zr-precursor 3

S1.3 Upscaling, downsizing & characterization 5

Section S2: Postsynthetic modification 7

S2.1 TG measurements & determination of siloxane amount 7

S2.2 NMR measurements 8

Section 3: Composite materials 10

S 3.1 Preparation and characterization 10

S 3.2 Mechanical Measurements 12

References 14

Section S1: General synthesis and parameter variation

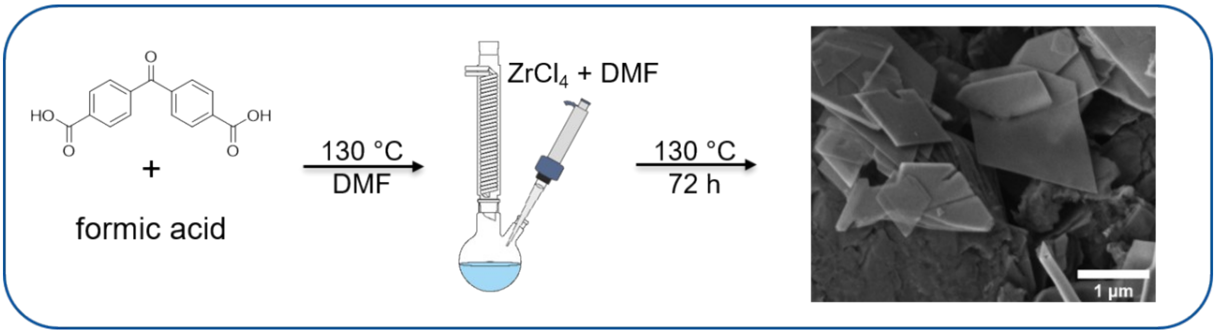

Figure S1: Synthesis route for the preparation of Zr-*bzpdc*-MOF monolayer thin micro flakes **1** via a formic acid modulated reflux synthesis and SEM image of the resulting product.

S1.1 Variation of reaction time & -temperature

Based on our previously published prescription for the synthesis of Zr-*bzpdc*-MOF single crystals,[1] a synthesis route for the preparation of ultrathin Zr-*bzpdc*-MOF micro flakes was developed. The most fundamental change is the altered reaction route under reflux instead of the previously solvothermal synthesis. Varying synthesis parameters include reaction time, synthesis temperature and the use of different Zr precursors. The use of additives was also tested. However, this did not lead to the desired results and is therefore not discussed in this paper.

Figure S2 shows PXRDs of reflux syntheses under variation of reaction time and temperature. Further parameters, such as concentrations and the Zr precursor, were initially selected analogous to the literature synthesis. It is obvious that crystalline products are obtained only from temperatures above 130 °C. For solvothermal syntheses of single crystals, temperatures of 120 °C were sufficient. Already after 6 h a crystalline product is present, which corresponds to the Zr-*bzpdc*-MOF. For synthesis times below 72 h, an increase in the background signal of the PXRDs at small °2Θ suggests an amorphous side product or an only partially crystalline product.

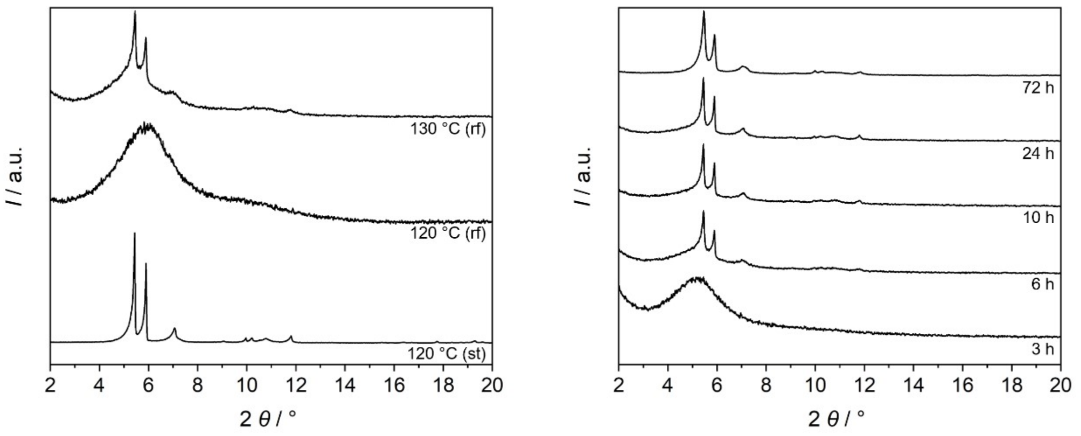

Figure S2: PXRDs of Zr-*bzpdc*-MOF reflux syntheses. Left: variation of Temperature with t = 20 h. Right: variation of reaction time with T = 130 °C. st = solvothermal, rf = reflux.

SEM images in Figure S3 support this assumption. In the products from syntheses with 10 h and 72 h reaction time, the Zr-*bzpdc*-MOF is present in both cases as 2-5 µm large and very thin rhomboids, which are partially intergrown. In the 10 h synthesis, however, a minor phase is still clearly visible. For this reason, syntheses were carried out at 130 °C and with a reaction time of 72 h in the following.

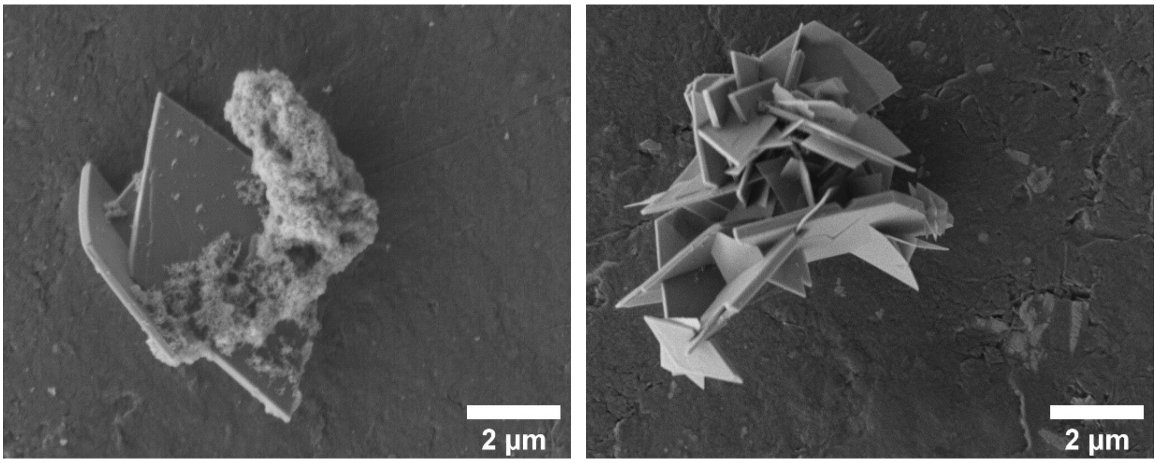

Figure S3: SEM images showing Zr-*bzpdc*-MOF micro flakes from syntheses at 130 °C after 10 h (left) and 72 h (right).

S1.2 Variation of Zr-precursor

In the next step, the influence of different Zr precursors on product formation was investigated. Besides zirconyl chloride octahydrate ($ZrOCl_2 \cdot 8\,H_2O$), zirconium(IV) chloride ($ZrCl_4$), zirconium(IV) hydroxide ($Zr(OH)_4$), zirconium(IV) acetate hydroxide ($Zr(OAc)_2(OH)_2$), zirconium(IV) oxynitrate hydrate ($ZrO(NO_3)_2{\cdot}H_2O$) and zirconium(IV) acetylacetonate ($Zr(Acac)_4$). The syntheses were carried out for 72 h at 130 °C under reflux. PXRDs in Figure S4 left show that crystalline products are obtained for all syntheses except when zirconium(IV) hydroxide is used.

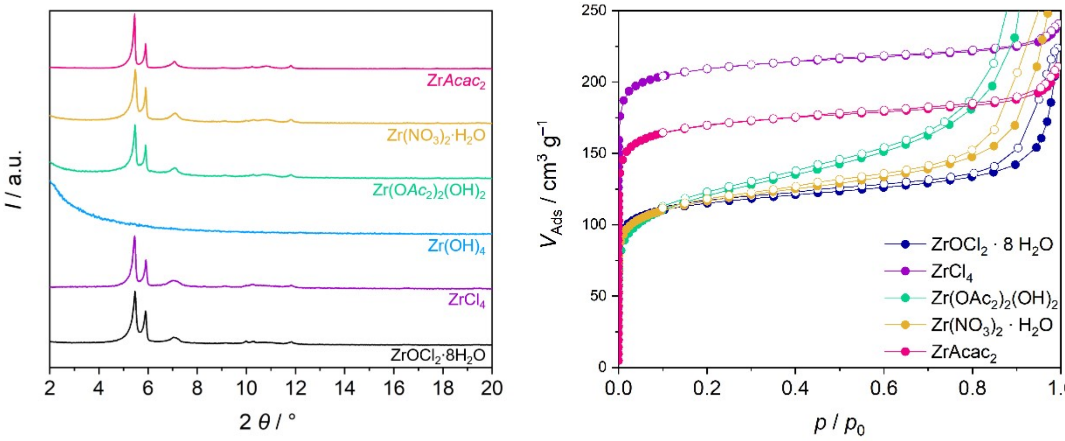

Figure S4: PXRDs (left) and argon-physisorption isotherms (right) of Zr-*bzpdc*-MOF reflux synthesis at 130 °C for 72 h. Different Zr precursors were used.

However, clear differences can be seen in the porosities determined via argon-physisorption (Fig. S4 right). Here, the highest value was obtained for the product from the synthesis with zirconium(IV) chloride with a BET area of 730 $m^2 g^{-1}$. For zirconyl chloride octahydrate, zirconium(IV) acetate hydroxide, zirconium(IV) oxynitrate hydrate, and zirconium(IV) acetylacetonate values of 400 $m^2 g^{-1}$, 350 $m^2 g^{-1}$, 400 $m^2 g^{-1}$ and 560 $m^2 g^{-1}$ were obtained. The differences are probably due to amorphous side products and lower crystallinity. The former can be seen in the SEM images (Figure S5) especially in the product of the $Zr(OAc)_2(OH)_2$ synthesis. Otherwise, in all syntheses the Zr-*bzpdc*-MOF is formed as very thin rhombohedral-shaped platelets.

Further characterization as well as upscaling were carried out in this work on the basis of the discussed results using $ZrCl_4$.

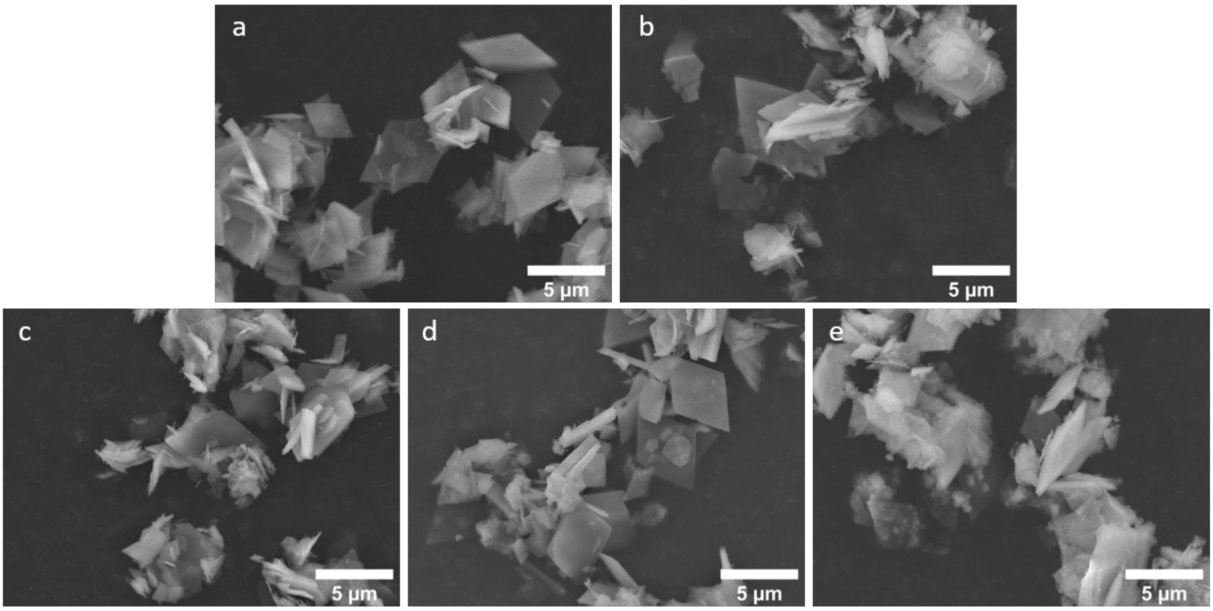

Figure S5: SEM images from syntheses with different Zr precursors with T = 130 °C and t = 72 h. a) zirconyl chloride octahydrate, b) zirconium(IV) chloride, c) zirconium(IV) acetate hydroxide, d)zirconium(IV) oxynitrate hydrate ($ZrO(NO_3)_2$ H_2O) and e) zirconium(IV) acetylacetonate.

S1.3 Upscaling, downsizing & characterization

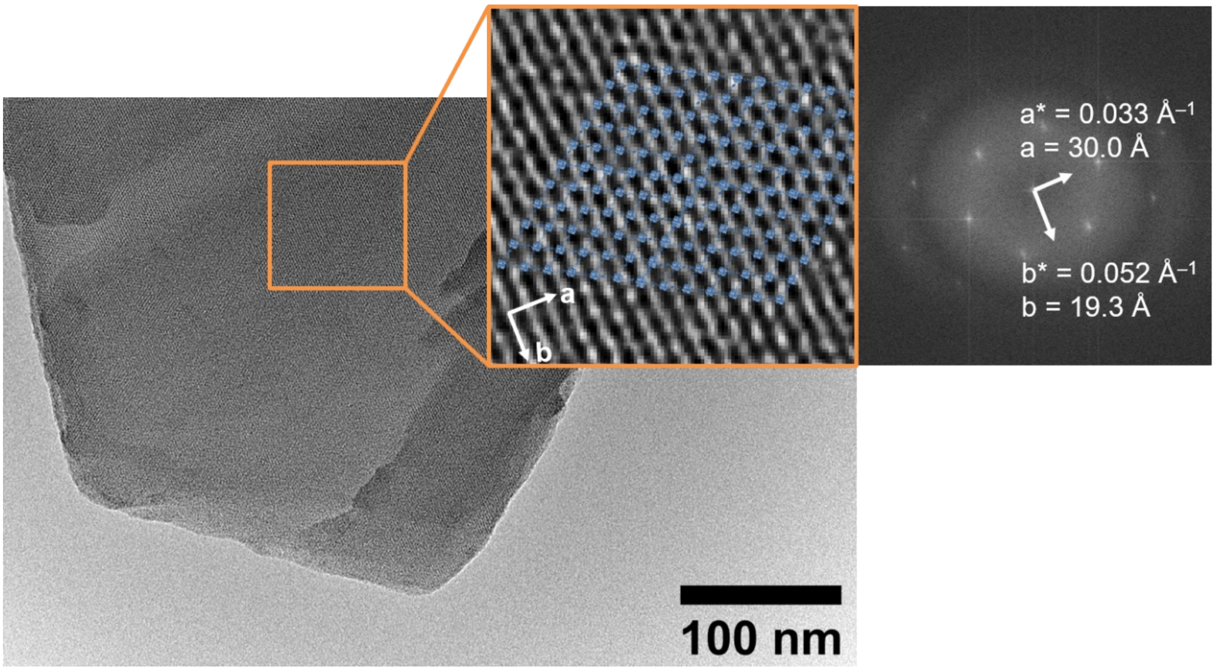

Figure S6: TEM images of Zr-*bzpdc*-MOF micro flakes. a) High resolution TEM with inserted structure and determination of lattice parameters; b) cross-section and c) top-view.

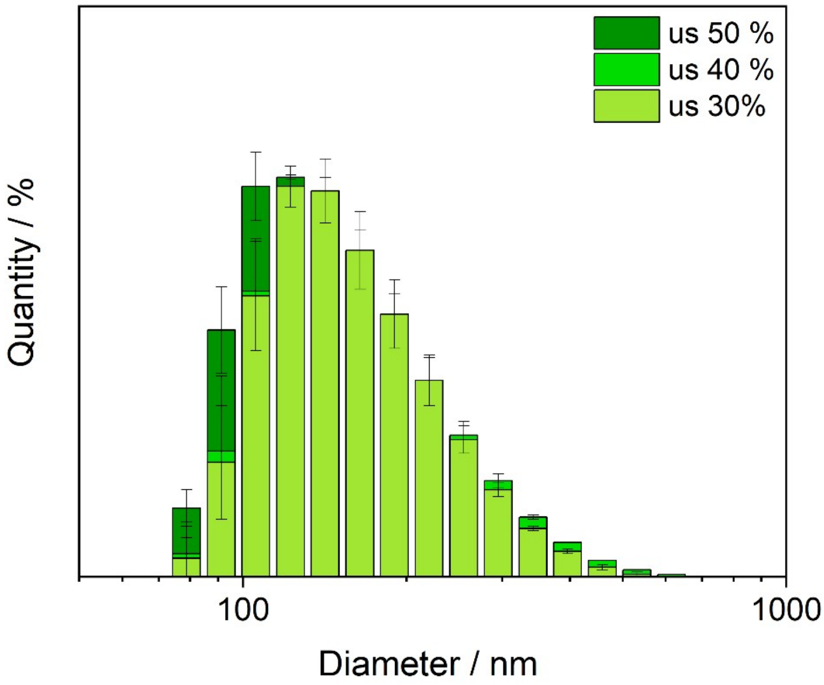

Figure S7: DLS measurements of Zr-*bzpdc*-MOF micro flakes **2** downsized via ultrasonication with 30 % to 50 % power.

The size distribution was determined via DLS measurements and is shown in Figure S7 for the ultrasound treatment at 30 %, 40 % and 50 % power. The smallest particles were obtained with 50 % power. However, the particles are assumed to be spherical in the DLS measurement. Since the micro flakes are in the form of thin platelets that are much larger in length and width, the values obtained are only of limited significance. Nevertheless, they can be used as a rough approximation for the sizes of the particles, since the particles in the SEM images have the same order of magnitude. With about 80 %, the DLS measurement for the majority of the particles yields a size between 90 and 200 nm. About 17.5 % of the micro flakes have a diameter of about 120 nm.

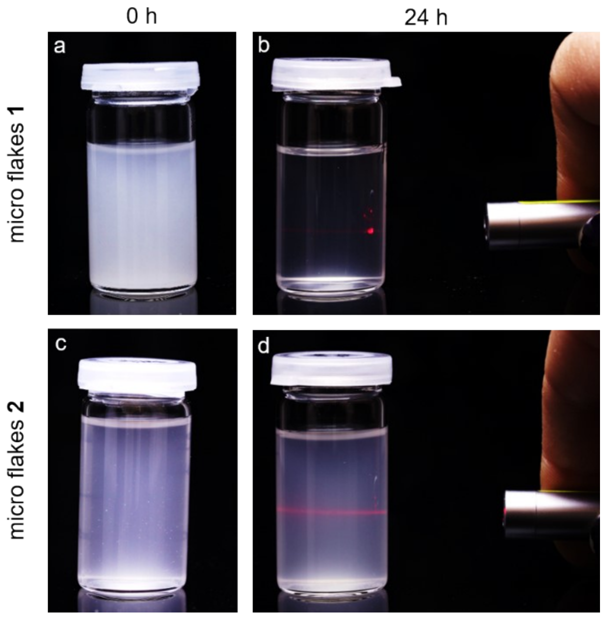

Figure S8: Dispersions of Zr-*bzpdc*-MOF micro flakes in acetone: a) micro flakes **1** direct after preparation and b) after 24 h; c) downsized flakes **2** direct after preparation and d) after 24 h. Different extend of light scattering (b, d) of a laser beam correlates with the stability of the dispersions after 24 h.

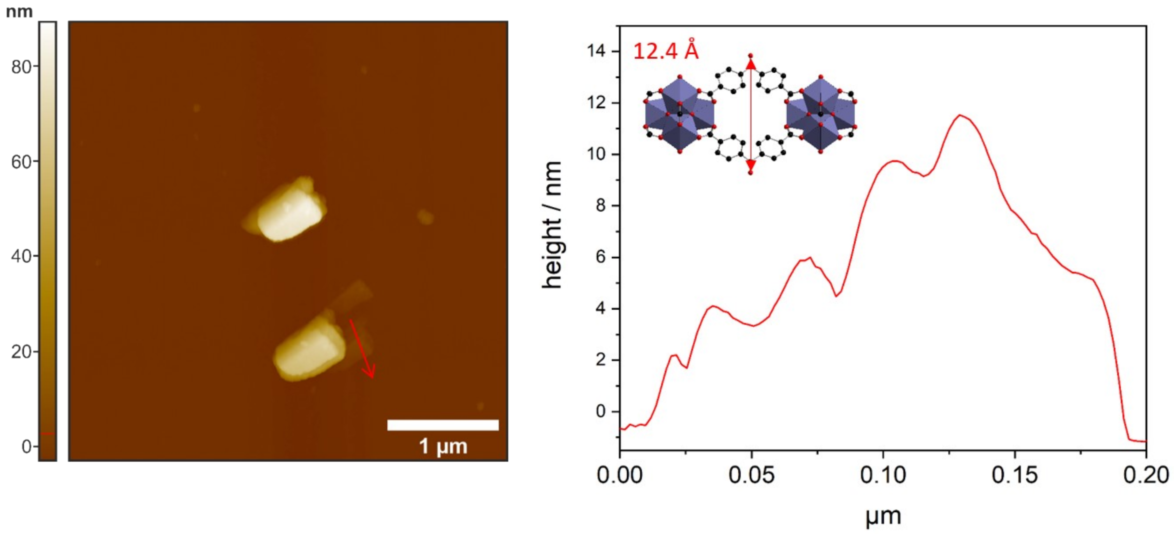

Figure S9: AFM-image (left) and height-profile (right) of Zr-*bzpdc*-MOF micro flakes after downsizing via ultrasonication in acetone.

Section S2: Postsynthetic modification

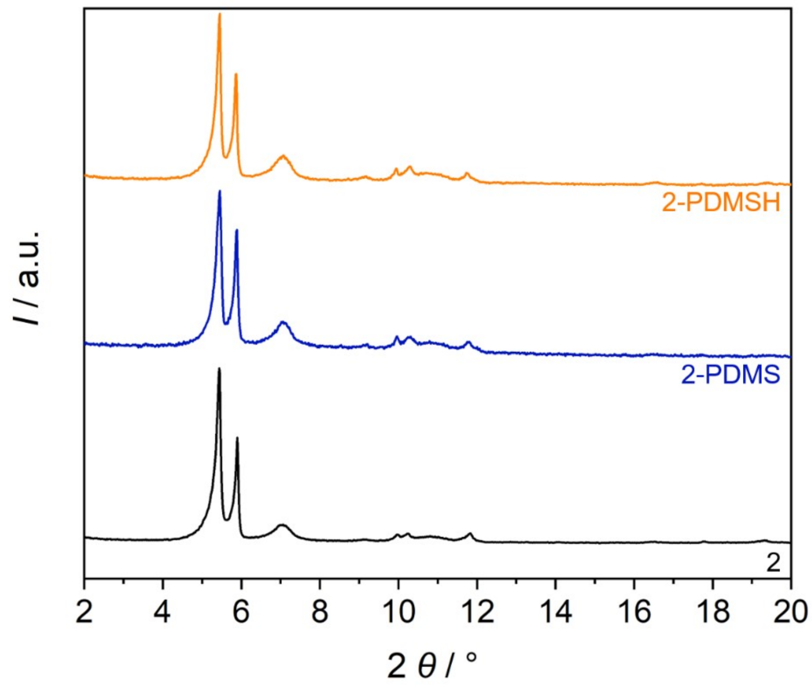

Figure S10: PXRDs of pristine Zr-*bzpdc*-MOF micro flakes **2** and after postsynthetic modification with PDMS (**2**-PDMS) and PDMSH (**2**-PDMSH).

S2.1 TG measurements & determination of siloxane amount

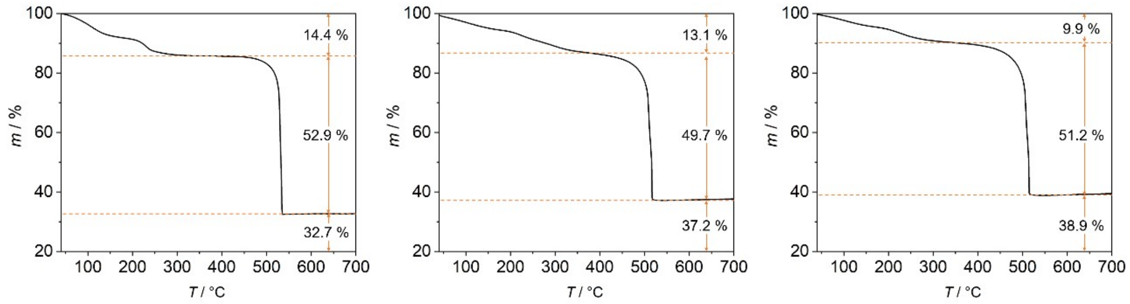

Figure S11: TG curves with calculated mass losses from left to right: **2**, **2**-PDMS and **2**-PDMSH.

TG measurements can be used to determine the amount of siloxane attached to the Zr-*bzpdc*-MOF micro flakes via PSM. The small mass difference between PDMS and PDMS-H is thereby neglected. Figure S11 shows the TG curves and calculated mass losses of pristine Zr-*bzpdc*-MOF micro flakes (**2**) and of flakes after modification with PDMS (**2**-PDMS) and PDMSH (**2**-PDMSH). Theoretical as well

as experimental mass fractions are summarized in Table S1. Experimental values are corrected by considering the guest molecules. With the aid of the theoretical composition of siloxane with the empirical formula $(CH_3)_2SiO$ and the experimentally determined composition of **2**, **2**-PDMS and **2**-PDMSH, the respective amounts of bound siloxane can be determined. An example calculation for **2**-PDMS is shown below.

Table S1: Theoretical mass fractions of Zr-*bzpdc*-MOF and siloxane as well as experimental composition of **2**, **2**-PDMS and **2**-PDMSH determined by TG measurements. Experimental data is corrected by subtracting guest molecules. Values used for the calculation are highlighted.

	Zr-*bzpdc*-MOF	$(CH_3)_2SiO$	**2**	**2**-PDMS	**2**-PDMSH
	theoretical			*experimental*	
organics	60.6	***19.0***	***61.8***	***57.2***	56.8
residue	39.4	81.0	38.2	42.8	43.2
% siloxane	-	-	-	***10.8***	11.6

The mass fractions can be determined with the aid of the corrected mass loss of organic matter of **2**-PDMS $w_{\mathbf{2\text{-}PDMS}}$ = 57.2 %. In addition, the corrected mass loss of organics in the pristine Zr-*bzpdc*-MOF micro flakes **2** $w_{\mathbf{2}}$ = 61.8 % and the theoretically calculated organic content of the silicone $w_{siloxane}$ = 19.0 % are needed.

$$
\begin{aligned}
w_{2-PDMS} &= x \cdot w_{siloxane} + (1-x) \cdot w_2 \\
&= x \cdot w_{siloxane} + w_2 - x \cdot w_2 \\
&= x \cdot (w_{siloxane} - w_2) + w_2
\end{aligned}
$$

By rearranging we get:

$$x = \frac{w_{2-PDMS} - w_2}{w_{siloxane} - w_2} = \frac{57.2\ \% - 61.8\ \%}{19.0\ \% - 61.8\ \%} = 0.108.$$

S2.2 NMR measurements

The NMR spectra of dissolved samples are shown in Figure S12. In all three spectra, the strongly pronounced signals of the aromatic H atoms in the *bzpdc* linker occur at 7.64 ppm and 7.79 ppm. These are doublets of triplets result from the coupling of the four H atoms. All other peaks are only very small in intensity compared to these doublets of triplets. The postsynthetic modification results in two additional signals at 7.30 ppm and 7.67 ppm for the spectra of the modified micro flakes with PDMS and PDMSH, which can also be assigned to the aromatic H atoms, but are shifted towards

lower ppm values. The cause of the high-field shift is the result of the reaction at the electron-withdrawing carbonyl group in the center of the linker molecule, which is converted into an electron-pushing OH-group and subsequently leads to a shielding of the H atoms in the neighborhood. The two additional signals are therefore an indication of successful modification. However, since the signals of the original *bzpdc*-linker are still present and predominant, only a modification of the outer surface can be assumed. In addition, PDMSH seems to be more reactive, the peaks at 7.30 ppm and 7.67 ppm are more intense, which suggests a higher degree of modification.

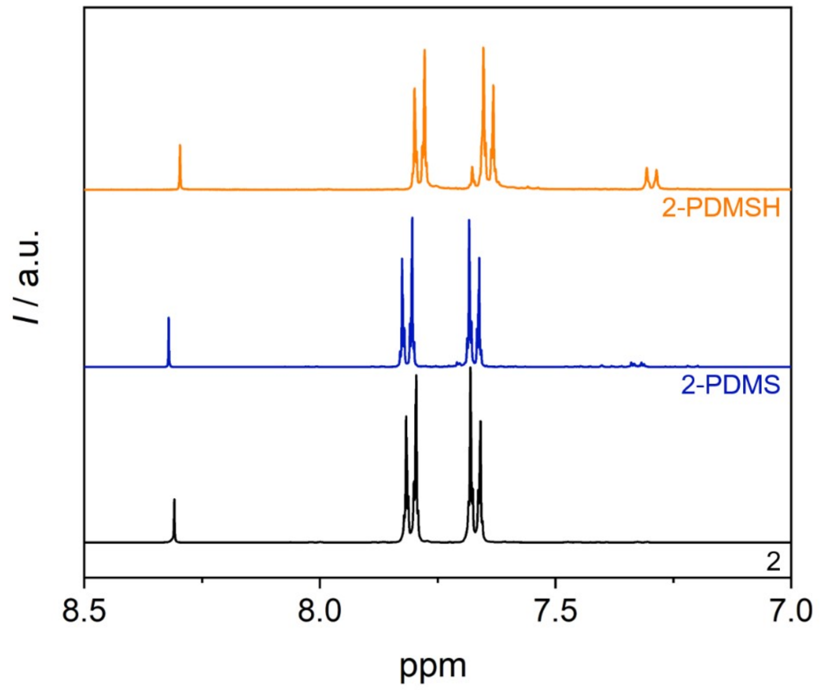

Figure S12: ^{1}H NMR spectra of pristine Zr-*bzpdc*-MOF micro flakes **2** and after postsynthetic modification with PDMS (**2**-PDMS) and PDMSH (**2**-PDMSH).

Section 3: Composite materials

S 3.1 Preparation and characterization

Table S2: Synthesis of the silicone elastomers with different mass fractions of modified and unmodified Zr-*bzpdc*-MOF micro flakes. Silicon components from Sylgard 184 are denoted as SilA and SilB.

MOF content [wt.%]	m_{MOF} [mg]	m_{SilA} [mg]	m_{SilB} [mg]
0	0	341	34
2.5	9	332	33
5	19	323	32
10	38	307	31
20	75	273	27
30	113	239	24

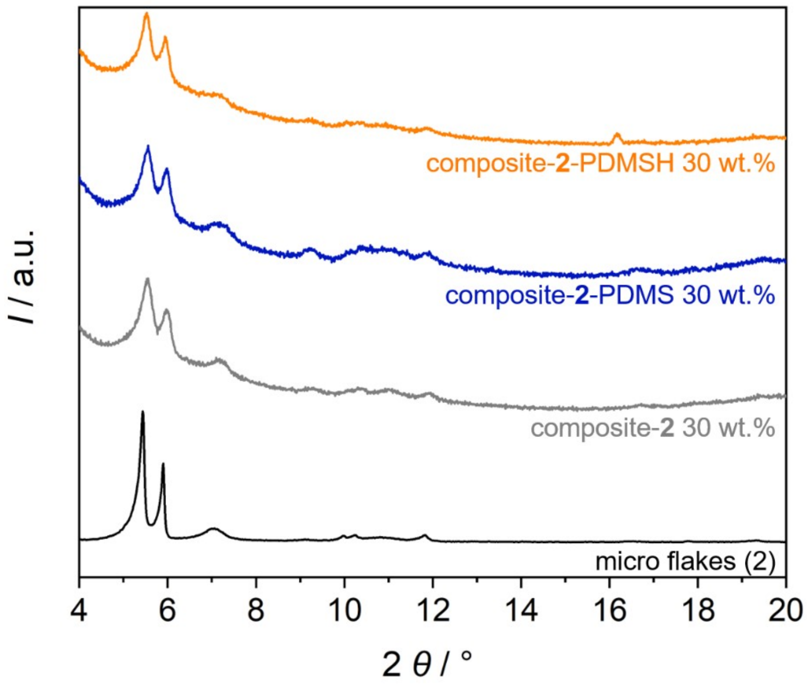

Figure S13: XRDs of polymer hybrid materials composite-**2**, composite-**2**-PDMS and composite-**2**-PDMSH with 30 wt.% MOF content. PXRD of powdered Zr-*bzpdc*-MOF **2** is shown as reference.

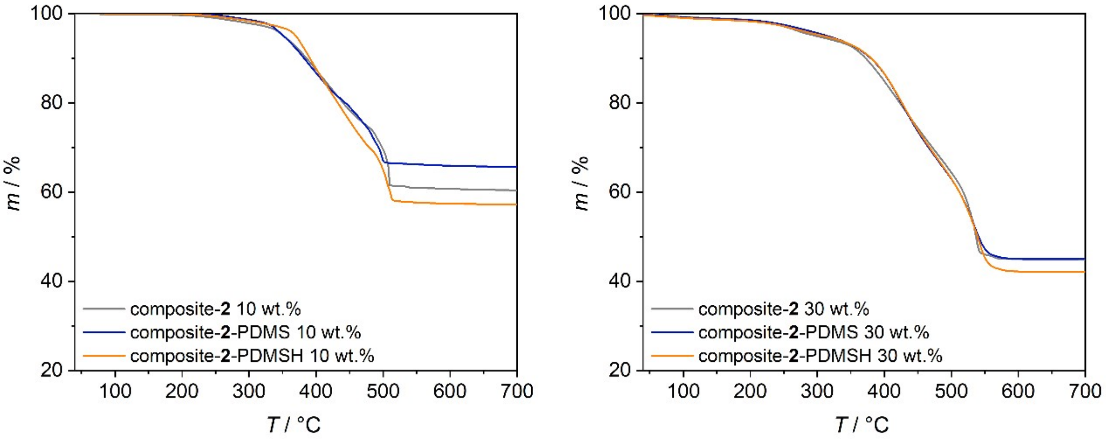

Figure S14: TG curves of composite materials with a MOF content of 10 wt.% (left) and 30 wt.% (right).

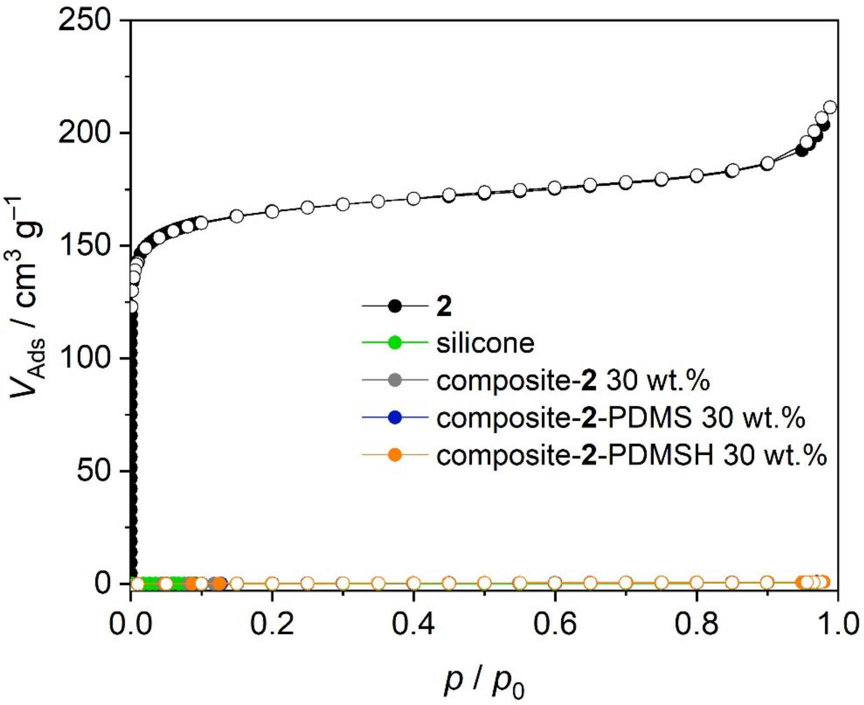

Figure S15: Argon physisorption isotherms (87 K) of composite materials with 30 wt.% MOF content and pure Zr-*bzpdc*-MOF micro flakes **2** for comparison.

S 3.2 Mechanical Measurements

Tensile tests were performed to determine the mechanical properties of the composite materials. Composite-**2** (with pristine Zr-*bzpdc*-MOF flakes) as well as composite-**2**-PDMS and composite-**2**-PDMSH (flakes modified with PDMS and PDMSH) with MOF content of 5 wt.%, 10 wt.% and 30 wt.% were investigated. Pure silicone materials (Sylgard 184) served as a control group. Three test specimens of each sample type were prepared and measured. Resulting stress-strain curves are shown in Figure S15. Young's modulus, ultimate tensile strength and elongation at break were determined for each specimen and are summarized in Table S2.

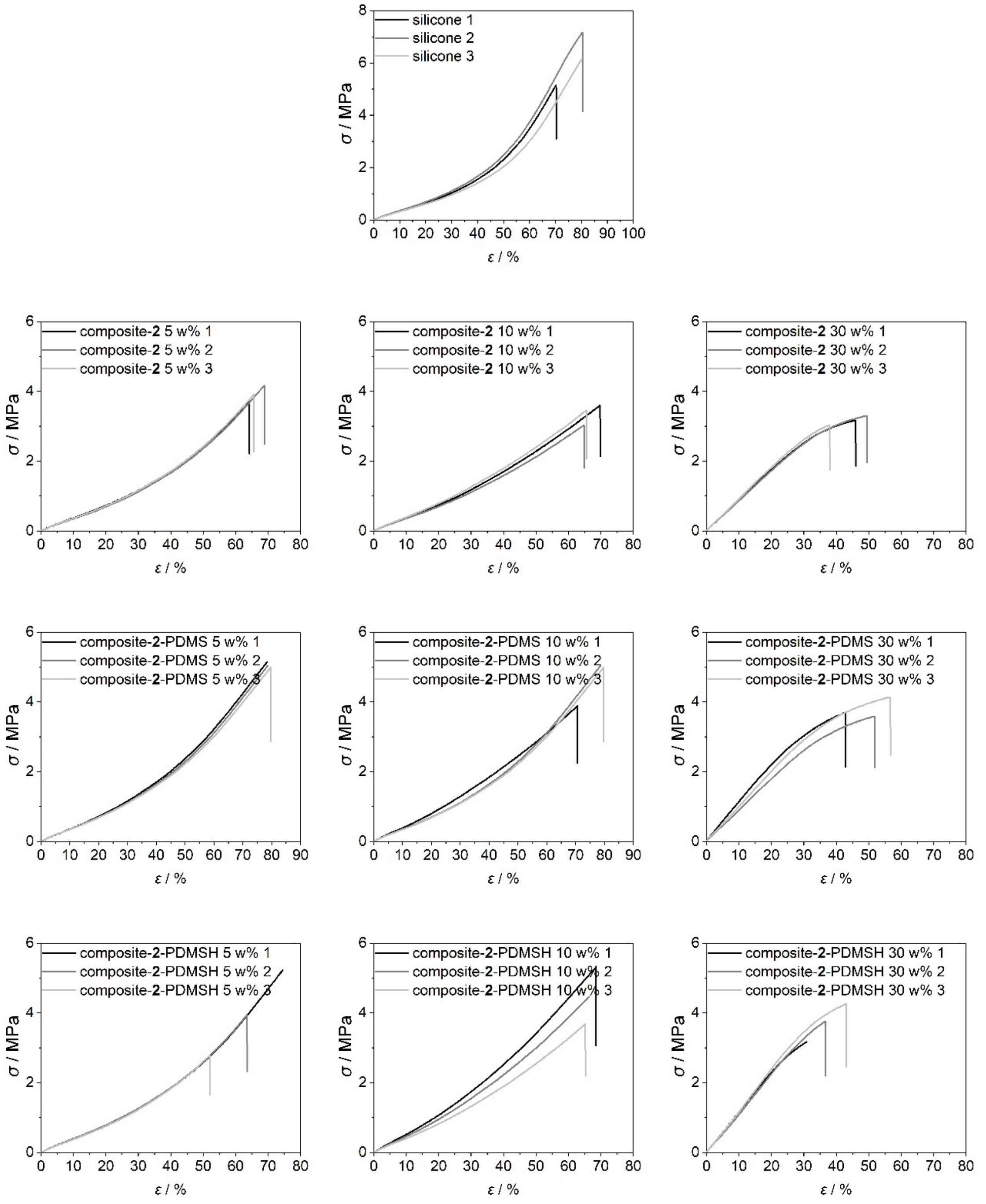

Figure S16: Stress-strain curves for pure silicon elastomer, and polymer hybrid materials with 5 wt.%, 10 wt.% and 30 wt.% MOF content.

Table S3: Calculated Young's moduli, fracture strain and breaking stress for polymer hybrid materials composite-**2**, composite-**2**-PDMS and composite-**2**-PDMSH with 5 wt.%, 10 wt.% and 30 wt.% filler content.

	Young's modulus / E	tensile strength / ε_B	elongation at break / σ_M
**Silicon**			
1	3.218	70.25	5.15
2	3.450	80.35	7.16
3	3.112	80.17	6.15
**composite-2 5 wt.%**			
1	3.634	64.14	3.71
2	3.421	68.85	4.17
3	3.503	65.55	3.91
**composite-2 10 wt.%**			
1	3.593	69.67	3.60
2	3.476	64.80	3.02
3	3.684	51.22	2.34
**composite-2 30 wt.%**			
1	8.670	45.81	3.17
2	8.432	49.27	3.30
3	8.983	37.85	3.03
**composite-2-PDMS 5 wt.%**			
1	3.499	78.40	5.14
2	3.484	78.59	5.03
3	3.387	79.61	4.99
**composite-2-PDMS 10 wt.%**			
1	3.767	70.68	3.89
2	4.370	58.73	3.56
3	4.655	73.65	5.16
**composite-2-PDMS 30 wt.%**			
1	11.235	42.75	3.70
2	8.740	51.80	3.58
3	9.810	56.54	4.13
**composite-2-PDMSH 5 wt.%**			
1	3.888	74.51	5.23
2	3.888	63.54	3.96
3	3.672	52.03	2.78
**composite-2-PDMSH 10 wt.%**			
1	5.103	68.44	5.30
2	4.497	66.45	4.47
3	3.974	65.21	3.69
**composite-2-PDMS 30 wt.%**			
1	11.449	30.87	3.17

2	10.580	36.74	3.75
3	11.361	43.04	4.26

References

1 A. Mohmeyer, A. Schaate, B. Brechtken, J. C. Rode, D. P. Warwas, G. Zahn, R. J. Haug and P. Behrens, *Chem. Eur. J.*, 2018, **24**, 12848.
